# Group I Intron as a Potential Target for Antifungal Compounds: Development of a *Trans*-Splicing High-Throughput Screening Strategy

**DOI:** 10.3390/molecules28114460

**Published:** 2023-05-31

**Authors:** Bastien Malbert, Virginie Labaurie, Cécile Dorme, Eric Paget

**Affiliations:** Early Discovery, Biochemistry Excellence, Centre de Recherche La Dargoire, Bayer SAS, 69009 Lyon, France

**Keywords:** RNA, self-splicing, group I, HTS, ribozyme, *Fusarium*

## Abstract

The search for safe and efficient new antifungal compounds for agriculture has led to more efforts in finding new modes of action. This involves the discovery of new molecular targets, including coding and non-coding RNA. Rarely found in plants and animals but present in fungi, group I introns are of interest as their complex tertiary structure may allow selective targeting using small molecules. In this work, we demonstrate that group I introns present in phytopathogenic fungi have a self-splicing activity in vitro that can be adapted in a high-throughput screening to find new antifungal compounds. Ten candidate introns from different filamentous fungi were tested and one group ID intron found in *F. oxysporum* showed high self-splicing efficiency in vitro. We designed the *Fusarium* intron to act as a *trans*-acting ribozyme and used a fluorescence-based reporter system to monitor its real time splicing activity. Together, these results are opening the way to study the druggability of such introns in crop pathogen and potentially discover small molecules selectively targeting group I introns in future high-throughput screenings.

## 1. Introduction

Because of its complex three-dimensional structures and its function in essential biological roles, RNA is an attractive target for small molecules discovery. However, the nature of RNA makes it a challenging target to address using small molecules. The negatively charged backbone leading to numerous non-specific interactions with cationic molecules, the putative structural redundancy related to the only four bases of RNA, the lack of knowledge of targetable RNA domains using small molecules, or difficulties in bypassing extremely abundant rRNA are contributing factors to the under-representation of RNA as small molecule targets [1,2,3]. Despite these difficulties, several successful examples of RNA targeting were reported within the last fifteen years, such as HIV-1 TAR RNA [4], HCV IRES IIA RNA [5], SMN2 RNA [6] or group IIB introns [7] for examples.

To date, successful RNA targeting is exclusively related to the pharma market, and to our knowledge no RNA-targeting molecule for agronomic use has been reported. If many protein targets are known in plant pathogens, new resistances appear regularly and finding a new mode of actions is becoming increasingly challenging. As we need to find new small molecules, targeting RNA could be interesting for plant diseases control, but only if we can set up a high-throughput screening system that is both reliable and inexpensive.

**Figure 1:**
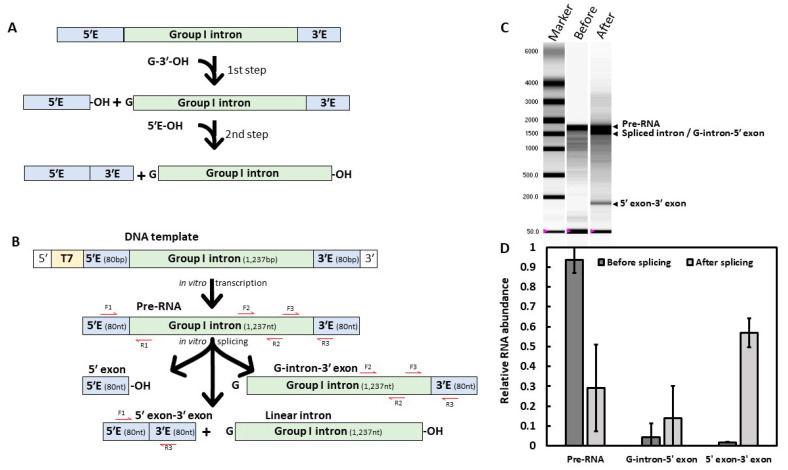
In vitro self-splicing of the *Fusarium oxysporum* cob group I intron. (**A**) Group I intron splicing mechanism. The 2-steps reaction starts with the addition of a guanosine (G-3′-OH) cofactor to the mix. (**B**) Experimental design. The *Fusarium* intron sequence was PCR amplified with 80 bp of border exons and a T7 promoter, then in vitro transcribed prior to the self-splicing reaction. Red arrows correspond to primers used for RT-qPCR. (**C**) RNA migration of reaction products, before (1) or after (2) the self-splicing reaction. Arrows represent the different splicing products obtained. Because of their similar length, spliced introns could not be distinguished from the intermediate product carrying the 3′ exon. RNA marker (M) sizes in nucleotides are indicated. Representative result of three independent experiments. (**D**) Quantitative Reverse Transcription PCR (RT-qPCR) of reaction products before and after splicing. Representative result of three independent experiments based on three technical replicates.

In this case, group I introns are a particularly attractive target. Present in fungi, including most of agro-relevant plant pathogens, they are much rarer in plants and almost absent in animals [8]. Group I introns rely on cofactors such as guanosine, suggesting a starting point for an RNA-related small molecule and a binding pocket that could be drugged [9]. Remarkably, these RNAs can self-splice from a precursor RNA (Pre-RNA), which occurs via a two-step transesterification mechanism. First, a guanosine cofactor binds the group I intron catalytic domain, and its hydroxyl group attacks the phosphorous atom at the 5′ splicing site, achieving the first step of the reaction (Figure 1A, 1st step). The guanosine is then attached to the 5′ terminus of the intron, and the 3′ exon exhibiting a free 3′ hydroxyl group is now able to attack the phosphorous atom at the 3′ splicing site, resulting in the removal of the intron from its primary transcript [10]. Moreover, it has been demonstrated that several group I introns can be artificially transformed into *trans*-splicing ribozymes by modifying their 5′ sequence [11,12,13,14,15]. These *trans*-acting ribozymes can be used to hydrolyze a substrate RNA, making them attractive for High-Throughput Screening (HTS) [11]. Although several group I introns have already been converted to *trans*-acting ribozymes, no ribozyme has been described from a major fungal plant pathogen to our knowledge.

In this paper, 10 group I introns from different subgroups and various plant pathogenic fungi were tested for their in vitro self-splicing activity. One intron from *Fusarium oxysporum* was able to achieve the two steps of its self-splicing in our conditions. This pathogenic fungus, responsible for the *Fusarium* wilt in a broad host range is known to cause important economic damages [16]. This intron is found in *F. oxysporum* clades 1 and 3, located in the *cob* transcript, and is relatively well conserved in many fungi [17]. As the cytochrome b protein is a component of respiratory chain complex III and the well-known target of Strobilurin-based (QoI) fungicides [18], the *F. oxysporum* group I intron in its transcript is a promising target efficiency wise, but with a different mode of action. In this work, we characterized this intron and studied its self-splicing activity. The results showed that the cis-intron self-spliced in vitro in conditions already known to promote splicing of *Anabaena* intron [19]. Based on this in vitro activity, we constructed a *trans*-splicing ribozyme and evaluated its efficacy in vitro to elaborate a low-cost, fluorescence-based assay that is amenable to HTS. These results suggest that this new ribozyme can be used in an HTS screen to discover new RNA-targeting small molecules. Finding sustainable and innovative modes of action remains important in small molecule research, and for this purpose, RNA targeting is a promising alternative. For agricultural applications however, molecules will still need to be relevant regarding agrochemical/fungal kinetics and expected selectivity, as it is currently done for protein targets.

## 2. Results

Ten group I candidates originating from different phytopathogenic fungi—*Botrytis cinerea* (*B.c.*), *Fusarium oxysporum* (*F.o.*), *Phakopsora pachyrhizi* (*P.p.*) and *Ustilago maydis* (*U.m*.)—were included in this study (Table S1). These introns were chosen among phytopathogenic fungi infecting economically relevant crops, in essential genes, for their high GC content, supposed to be positively correlated with in vitro self-splicing efficiency [20]. To investigate whether these introns could achieve in vitro self-splicing, a PCR amplification was performed keeping 80 bp of their 5′ and 3′ exons and adding a T7 promoter in 5′ (Figure 1B), which was subsequently used as a DNA template for in vitro transcription. Self-splicing was then monitored indirectly using RT-qPCR (Figure 1B) or directly with an automated electrophoresis system after RNA denaturation (Figure 1C). Of all candidates, seven did not show any self-splicing activity (Figure S1). Two introns, *B.c.* cob (4) and *P.p.* cob (1) seemed to undergo only partial self-splicing activity (Figure S1). This result was reproduced in three independent experiments and confirmed through RT-qPCR (Figure S2) with an enhanced G-intron-3′ exon content detected after the reaction.

The *Fusarium oxysporum* cob (1) intron performed the two self-splicing steps. To analyze the potential of this intron to be used as a *trans*-splicing ribozyme, we proceeded as follows. First, the intron was characterized, and a secondary structure was constructed to convert the ribozyme 5′ terminus into a *trans*-acting ribozyme with its RNA substrate [12,21]. Then, the in vitro *trans*-splicing activity of the *F. oxysporum* ribozyme was assessed and an HTS-compatible approach was developed.

### 2.1. In Vitro Self-Splicing Activity of Fusarium Cob Intron

The *F. oxysporum* UASWS AC1 cob gene is composed of two coding sequences of 392 and 779 bp, interrupted by a group I intron of 1237 bp containing 27% GC. We investigated its in vitro self-splicing activity. We could not determine if the band below the pre-RNA corresponded to the linear intron, the intron-3′ exon intermediate or both (Figure 1C). Additionally, this method did not allow us to evaluate the splicing efficiency of *Fusarium* intron. To estimate and confirm *Fusarium* intron self-splicing activity, purified splicing reactions were reverse transcribed with a specific primer located within the 3′ exon and the resulting cDNAs were quantified using RT-qPCR (Figure 1B–D). Either excised introns or 5′ exons could not be monitored due to the absence of 3′ exon preventing them from being retrotranscribed. After one hour, approximately 70% of the pre-RNA was spliced, either in G-intron-3′ exon or in 5′ exon-3′ exon (Figure 1D). Using primers located within the exons, the 5′ exon-3′ exon cDNA was amplified, purified and its sequence was confirmed using Sanger sequencing (Figure S3).

### 2.2. Trans-Splicing Ribozyme Design

**Figure 2:**
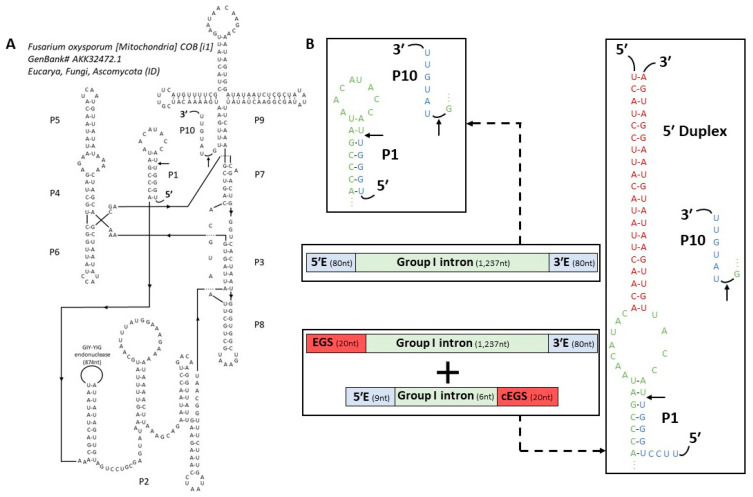
Secondary structure prediction. (**A**) Predicted secondary structure of *Fusarium oxysporum* cob group I intron. Common Group I domains are designated P1 to P10, and 5′-3′ ends are indicated. Arrows correspond to splice sites. The site of GIY-YIG endonuclease insertion is indicated in domain P2. (**B**) P1 domain comparison between the group I native intron (top) and the ‘classical designed’ *trans-acting* ribozyme (bottom). Schematic diagram of base-pairing between the ribozyme and the associated RNA target. Exon sequences are in blue, intron sequences in green and additional sequences EGS and complementary EGS (cEGS) are in red.

In order to convert group I into a *trans*-splicing ribozyme, we predicted the secondary structure of the intron (Figure 2A) based on the prediction made for a homologous intron from *Podospora anserina* (CRW; Figure S4). Using RNAweasel [22] and based on P7/P7′ sequence alignment [8], this *Fusarium* intron appears to be part of the ID subgroup, more specifically to subgroup ID1 according to P3, P4, P6 and P8 domain alignments [23]. The sequence of a putative GIY-YIG endonuclease was found in the P2 domain, explaining the unusual length of the intron. However, as the start codon was not well defined the separation between the P2 domain and the putative ORF remained unclear. The proposed delimitation is based on both best base pairing within the P2 domain and the longest ORF predicted (275 AA) and it should be noted that as such, the fourth RNA codon (AUA) may actually be the start [24]. The internal guide sequence (IGS) was predicted to start at the 7th nucleotide after the 5′ splice site and was supposed to be 8-nt in length (Figure S5). The secondary structure prediction was then used to design the *trans*-acting ribozyme and its substrate (Figure 2B).

Several group I introns have already been converted into *trans*-splicing ribozymes [12,13,14,15,21], offering numerous design opportunities. The “classical design” from *Tetrahymena* ribozyme was arbitrarily chosen, with an extended guide sequence (EGS) to ensure an optimized ribozyme-substrate hybridization [12]. This resulted in a 5′ Duplex, a structure allowing specific hybridization between the substrate and the *trans*-splicing ribozyme while minimizing the free energy of unfolding and releasing the IGS [25]. An extra 4-nt were added to this EGS with the second half of the P1 domain, keeping the pattern proposed by Köhler et al. (1999) [12], followed by P2 to P9 domains and 80 nt of the 3′ exon (Figure 2B). The substrate was composed of the 9-nt located before the 5′ splice site, the first half of the P1 domain and the reverse-complement of the 5′ Duplex sequence. We aimed to stay as close as possible to the P1 domain from *Fusarium* so that the structure of the ribozyme remains close to that of the *cis*-intron. Doing so, the computed RNA binding energy was close to −6 kcal/mol, which is supposed to be positively correlated with *trans*-splicing efficiency [25].

**Figure 3:**
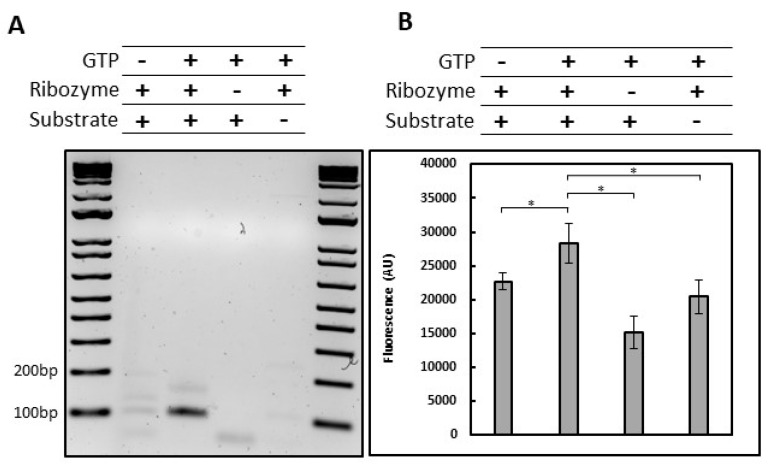
In vitro *trans*-splicing activity of *Fusarium* ribozyme. (**A**) Amplification of the 5′ exon-3′ exon ligation product by RT-PCR using 3′ exon specific primer for the RT, 5′ exon and 3′ exon specific primers for the PCR. The 5′ exon primer is an 18-bp oligonucleotide with only 9-bp annealed to the 5′ exon. The RT-PCR product corresponds to a 98-bp amplification (9-bp for the 5′ exon primer extension, 9-bp for the 5′ exon and 80-bp for the 3′ exon). Splicing reactions were performed with (+) or without (-) GTP, *Fusarium* ribozyme or substrate. (**B**) Quantitation of the 5′ exon-3′ exon ligation product formed during *trans*-splicing by a molecular beacon. Splicing reactions were performed with (+) or without (-) GTP, *Fusarium* ribozyme or substrate. Error bars correspond to standard deviation. Error bars correspond to standard deviation. Representative experiment of three independent experiments, the stars correspond to the *p*-value of a multi-factor ANOVA followed by a Bonferroni correction (* *p* < 0.01).

After in vitro transcription, the *Fusarium* ribozyme exhibited in vitro *trans*-splicing activity as shown by amplifying the 5′ exon-3′ exon splice product (98-bp) using an RT-PCR approach. This result was only observed when the substrate, ribozyme and GTP were added together to the reaction mix, suggesting specific amplification (Figure 3A and Figure S6). A faint, but still visible band was also observed around 100-bp for the condition without GTP. As the ribozyme was produced by in vitro transcription, we may still have a very low amount of GTP with the ribozyme despite the purification steps. Another explanation would be that in absence of GTP, a less efficient hydrolytic cleavage happened [26,27].

Then, a quantitation of *Fusarium* ribozyme *trans*-splicing product was achieved using a molecular beacon [28]. The assay is based on a beacon composed of a fluorophore in 5′, a quencher in 3′ and a loop, exhibiting fluorescence only when hybridized with its target. A slight but consistent increase in fluorescence was observed in the +GTP condition, correlated to the detection of the 5′ exon-3′ exon *trans*-splicing product (Figure 3B). Together, these results confirmed that *Fusarium* ribozyme can be used in a high-throughput screening with a proper readout.

### 2.3. HTS-Compatible Fluorescent Reporting System

**Figure 4:**
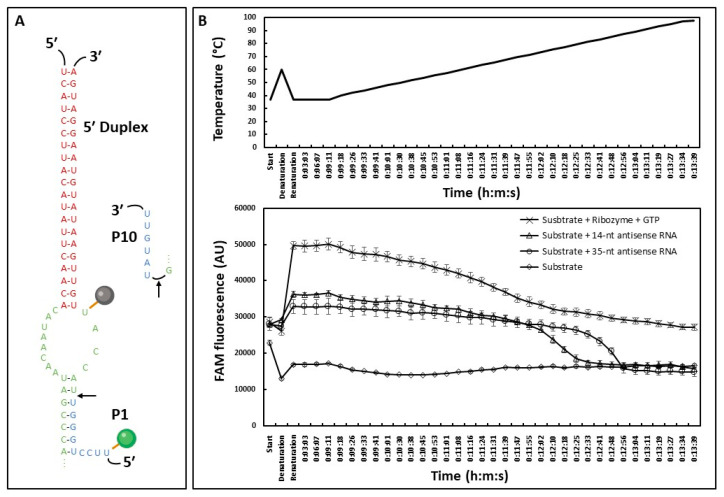
Fluorescence-based assay exploring the different signal components. (**A**) Substrate used in this experiment. The substrate is composed of a FAM fluorophore in green (maximum excitation: 495 nm; maximum emission: 515 nm) and a DABdT quencher in grey (maximum absorbance: 475 nm); 13-nt separate the quencher from the fluorophore. (**B**) Fluorescence-based assay. The 35-nt substrate was either mixed with the ribozyme and GTP for a splicing reaction (cross), with a 35-nt antisense RNA (circle), a 14-nt antisense RNA (triangle) or alone (diamond). After 2 min at 37 °C to assess initial fluorescence (start), samples were heated up to 60 °C for 2 min (denaturation) and slowly cooled down to 37 °C during 1 h (renaturation). Samples were then kept at 37 °C for 10 min and the temperature was increased by 2 °C every 7 to 8 s with fluorescence measurements. Representative experiment based on three technical replicates. Error bars correspond to standard deviation.

To identify new antifungal compounds selectively targeting group I introns splicing, we developed a high-throughput fluorescence assay monitoring the *Fusarium* ribozyme *trans*-splicing activity. Based on what had been accomplished on ai5γ group II intron from *Saccharomyces cerevisiae* [7], we conjugated a fluorescence quencher and a fluorophore on opposite sides of the 5′ splice site to monitor an enhanced fluorescence signal when the ribozyme cleaves the substrate, corresponding to the first step of the self-splicing reaction (Figure 4A).

**Figure 5:**
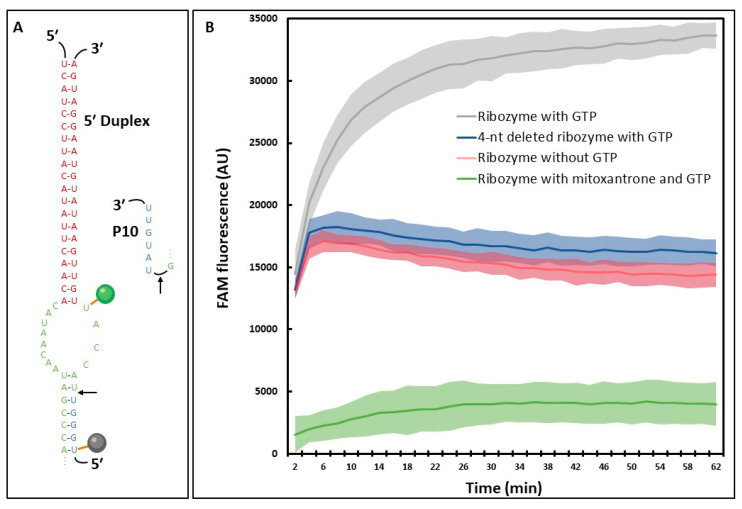
Fluorescence-based HTS-ready assay to screen inhibitors of *Fusarium* intron. (**A**) P1 and 5′ Duplex domains of the fluorescently labeled substrate and the associated ribozyme. The quencher (Iowa Black^®^ Quencher FQ, maximum absorbance: 531 nm), and the FAM fluorophore are represented by a black and a green circle, respectively. (**B**) Fluorescence measurement of *trans*-splicing activity with (grey) and without (red) GTP, using the 4-nt deleted ribozyme with GTP (blue) or with the addition of 10 µM of mitoxantrone (green). Shaded areas correspond to standard deviations. Representative experiment of three independent experiments.

To test if the signal corresponded fully or partially to substrate splicing, we compared the fluorescence obtained from the substrate with the ribozyme to the fluorescence obtained from the substrate and antisense RNAs (asRNAs) that would then be related to hybridization and not splicing (Figure 4B). At 37 °C, the fluorescence signal obtained with asRNAs corresponded approximately to half the signal observed with the ribozyme. We assumed that it was due to the formation of an RNA-RNA duplex able to pull the fluorophore away from the quencher, thereby enhancing the fluorescence signal. Using a melting curve approach, a clear drop in fluorescence intensity was observed for asRNAs conditions upon increasing temperatures (i.e., RNA-RNA dimer de-hybridization), down to the fluorescence level of the substrate alone. This melting curve also exhibited a shift in temperature depending on the asRNA length, whereas the fluorescence obtained with the ribozyme remained stable at high temperatures. All put together, these data suggested that only part of the signal was splicing-related and highlighted the low quenching efficiency of this substrate represented by a high fluorescence level of the substrate alone. To enhance the splicing-specificity of the signal, we chose to shorten the substrate in 5′ by suppressing 4 nt, which brought the quencher closer to the fluorophore and thus, enhanced the sensitivity of our system (Figure 5A). A new quencher was also used and proved to be a better match with our fluorophore (Figure S7, see ‘Materials and Methods’ Section 4). Fluorophore and quencher were tested at different positions within the substrate to compare the quenching efficiency. Although it was tempting to use a fluorophore and a quencher surrounding the splicing site (Figure S7C), the splicing efficiency of the reaction seemed to be reduced, and eventually a substrate with similar positions compared to the first one tested was chosen (Figure S7B). Additionally, the splicing-related fluorescence was assessed by comparing the signal with and without GTP to account for the hybridization-related fluorescence (Figure 5B, grey and red curves; Figure S8), showing a two-fold increase in presence of GTP. This increase is not seen with a 4-nt deletion in the P7 domain of the ribozyme in presence of GTP (Figure 5B, blue curve; Figure S9), suggesting a splicing-related increase in the fluorescence signal.

We then proceeded to test the effect of a known small molecule on the system. Since there are no specific group I inhibitors to our knowledge, we tested mitoxantrone, a non-specific RNA binder. Mitoxantrone was found earlier to inhibit H.C. LSU group II intron splicing in vitro [28] and bind stem-loop RNAs [29,30]. A 10 µM dose of mitoxantrone was chosen based on Omran et al. (2022) [28], corresponding to a complete inhibition of H.C. LSU group II intron splicing in vitro (Figure 5B, green curve). The observed fluorescence appeared to be lower than the condition without GTP, suggesting a fluorescence quenching effect and/or blocking the substrate into a stem-loop structure thus preventing the ribozyme-substrate hybridization.

## 3. Discussion

### 3.1. A Low GC Content Group I Intron Able to Self-Splice In Vitro

In this work, we aimed to develop an HTS-compatible assay to find small molecules targeting group I introns. We tested the self-splicing activity of 10 group I introns in fungi of agronomic interest. To increase our chances of finding a proper candidate, we have tried to follow recommendations from previous work, such as >35% GC, which is described to be positively correlated with in vitro self-splicing efficiency [20]. However, it was not possible to reach it for most of our candidates, as the average mitochondrial GC content in fungi is close to 25%, 10 points lower compared to Plants and Animals [31,32]. Based on this information, we selected candidates with the highest GC content, mostly focusing on subgroups IA and ID, often seen as in vitro self-splicing introns [20]. Microcapillary electrophoresis was first used to assess self-splicing activity. Despite the impossibility to distinguish between fragments of similar sizes and to precisely quantify splicing products, its ease of use makes the screening of numerous candidates possible in a short amount of time. On the other hand, RT-qPCR has emerged as a complementary approach to microcapillary electrophoresis, with accurate quantification of most RNA fragments [7,33]. In our non-optimized conditions, 9 out of 10 introns did not show in vitro self-splicing activity. Among them, we believe *B.c.* cob (4) and *P.p.* cob (1) accumulate the G-intron-3′ exon form, corresponding to the first step of the self-splicing reaction, but the 5′ exon-3′ exon is neither detected by microcapillary electrophoresis nor by RT-qPCR. It is reasonable to assume that different factors such as buffer composition, arbitrary 5′ and 3′ exon lengths, incubation time or annealing conditions [20] may not be suitable for all our candidates. Therefore, it should be important to test many other conditions to see whether these nine introns can self-splice in vitro.

One, however, was found to achieve the two steps of the self-splicing reaction. It is a cob intron from *Fusarium oxysporum* (*F.o.* cob (1)), harboring the lowest GC content (27%, 33% excluding the ORF) of all the surveyed group I introns in this work. Apart from the *Saccharomyces cerevisiae* bI5 intron (16% GC) which has been described to have low self-splicing [20,34], *F.o.* cob (1) has the lowest GC content of introns that has been shown to efficiently self-splice in vitro [34,35,36,37,38,39,40,41,42,43,44,45,46,47,48,49,50,51,52,53,54,55,56].

### 3.2. Comparison between Fusarium Oxysporum and Neurospora Crassa cob (1) Intron

Aiming to develop a screening system for small molecules that can block the splicing of this intron, it is important that the in vitro and in vivo structures of *F.o.* cob (1) are as similar as possible. This raises the problem of intron-binding proteins, which would modify its structure and therefore alter potential interactions with small molecules we may observe in vitro [57,58,59,60,61]. In this work, a strong argument in favor of an absence of protein in vivo is the capacity of *F.o.* cob (1) intron to self-splice in vitro near physiological temperature, suggesting that no high enthalpy activation is needed [60,62].

As we are not aware of any additional data on this intron, we compared *F.o.* cob (1) to the same intron in *Neurospora crassa*, another filamentous fungus. These two introns of the same subgroup have many commonalities, such as their low GC content (27 and 29% for *F.o.* and *N.c.*, respectively), the presence of an ORF encoding a GIY-YIG, a complex P9 domain as well as the absence of P5abc domains, or the putative absence of circular intron after self-splicing in vitro [36,51]. In *N. crassa*, Wallweber et al. showed that the *cyt-18-1* mutant has a defect in the splicing of the cob (1) intron. How the CYT-18 protein, a tyrosyl-tRNA synthetase, facilitates cob (1) splicing in vivo is not clear, since no direct interaction or effect on in vitro splicing efficiency with the CYT-18 protein was observed [51]. The *Neurospora* cob gene also contains a second intron 94 bp downstream of the cob (1) intron, which has been shown to interact with CYT-18 and its splicing efficiency is also affected in the *cyt-18-1* mutant. This second intron can also be found in some strains of *Fusarium* clade 1, but not in clades 2 or 3 such as the UASWS AC1 strain used in this work [17]. Rather than a direct role of CYT-18 in *Neurospora* cob (1) intron splicing, these data may suggest the existence of a co-dependent mechanism when two introns are found in close vicinity with one of the two having a defect that affects the splicing of the second [63,64]. Additionally, tyrosyl-tRNA synthetases such as CYT-18 are known to have activity in splicing group I introns only in *Pezizomycotina*, a subphylum that includes *Neurospora* but not *Fusarium* species [65]. Altogether, this makes it very unlikely that the splicing of *F.o.* cob (1) intron is protein-dependent, making it an interesting target for RNA-targeting small molecules discovery using a protein-free in vitro reporter system.

### 3.3. A high-Throughput Fluorescent Assay Based on Trans-Splicing Activity to Find New Antifungal Molecules

It was previously shown that small molecules targeting introns could be found using an in vitro *trans*-splicing approach [7]. Using the well-characterized ai5γ group II intron from *S. cerevisiae*, the authors created the D135 ribozyme, able to catalyze the cleavage of a specific RNA substrate conjugated with a fluorophore and a fluorescence quencher on opposite sides of the splicing site. Here, we applied the same strategy to develop an assay aiming at screening small molecules libraries in a high-throughput manner: this required us to predict its secondary structure, create its corresponding *trans*-splicing ribozyme, and develop a fluorescent reporter assay.

Applying a well-described strategy [12], *Fusarium trans*-splicing ribozyme and substrate were constructed keeping the G·U base-pair adjacent to the 5′ splice site. The P1 helix is opened to allow the addition of an extended guide sequence (EGS) to ensure ribozyme-substrate specificity and affinity, while trying to keep the same structure and sequence for a proper P1 and P10 overlap upon the internal guide sequence. Since only this construct has been tested, its activity can probably be improved, with a different EGS for example [21] or point mutations improving its folding [7].

To find antifungal molecules, an in vitro reporter assay was developed for high throughput screening, eliminating PCR-based approaches. Two fluorescence-based strategies were considered: the recently published beacon assay [28] and the ai5γ approach [7] used in this work. The main advantage of the beacon assay is that it does not require a *trans*-splicing approach to work, reducing the level of complexity of the approach. However, it can only be used for an endpoint reading while the ai5γ approach allows kinetics. An unexpected result in the ai5γ approach is the fluorescence increase that we think to be caused by RNA-RNA interaction. With a fluorophore and a fluorescence quencher 9-nt apart on the substrate, it seems that the base pairing with the ribozyme or a complementary RNA is sufficient to trigger a fluorescence signal. This was demonstrated using asRNAs (Figure 4B) but also a ribozyme carrying a deletion in the P7 domain displaying the same level of fluorescence as the native ribozyme without GTP (Figure 5B). However, the hybridization-related fluorescence was not observed with a fluorophore and a quencher separated by less than 3-nt neither in Fedorova et al. nor in this study (Figure S7C). This issue can be counterbalanced with proper controls such as the use of a ribozyme carrying a deletion within the P7 domain (Figure 5B), a comparison with and without MgCl_2_, EDTA or GTP for example. It is also possible to take advantage of this additional signal during a HTS. Several HTS assays based on fluorescence reported challenges, such as small molecules exhibiting autofluorescence or inversely, quenching activities, unrelated to the biological assay and leading to an important rate of false positives and negatives [66]. Therefore, having a well-defined upper and lower limit of the *trans*-splicing system could be primordial to identify proper inhibitors of group I introns. Using conditions with and without GTP allows us to know the minimum and maximum fluorescence related to the splicing activity of the ribozyme. If the addition of a small molecule to the reaction enhances the fluorescence higher than the maximum expected, it can be linked to a molecule improving splicing efficiency, exhibiting autofluorescence or affecting the quencher. On the other hand, a small molecule inducing a fluorescence signal lower than the expected minimum may be linked to an interaction with the fluorophore, a quenching activity, or a substrate-ribozyme interaction inhibition. Knowing these upper and lower values would allow one to detect part of non-specific and unwanted small molecules in a primary screening without further experiments, as we observed with the mitoxantrone fluorescence signal lower than the -GTP condition or the deletion mutant (Figure 5B). Based on this result, mitoxantrone was not shown to be a useful tool compound for *Fusarium trans*-ribozyme assay, either because of a fluorescence quenching activity or a substrate-ribozyme interaction inhibition.

In this work, we confirmed that a *trans*-ribozyme can be designed from an agriculture-relevant Group I intron and its activity monitored in a fluorescent assay. This will lead to further high-throughput screening of small molecules and for that purpose, specificity and selectivity will need to then be tackled still in vitro and also in vivo. Further experiments are necessary to acquire a better fundamental knowledge of *Fusarium* cob intron, to improve its splicing efficiency in vitro and to compare with self-splicing introns from the same subgroup or not, and from other pathogen organisms. This will be facilitated by the increasing interest of the scientific community in RNA structure and its relationship with small molecules and by the development of new techniques and tools, such as the recent public database RiboCentre [67].

## 4. Materials and Methods

### 4.1. Intron Selection

Mitochondrial introns from *Botrytis cinerea* (KC832409.1), *Fusarium oxysporum* (KR952337.1), *Phakopsora pachyrhizi* (GQ332420.1) and *Ustilago maydis* (DQ157700.1) were listed from the Organelle Resources (available from https://www.ncbi.nlm.nih.gov/genome/organelle/ accessed on 1 February 2020) using RNAweasel [22].

### 4.2. RNA Production

#### 4.2.1. Construct Preparation

All the introns surveyed are resumed in Table S1 with primers listed in Table S2. Briefly, a construct consisted in a T7 polymerase promoter followed by one guanosine residue, the last 80 bp of the 5′ exon, the intron, and the first 80 bp of the 3′ exon of the gene. PCR amplification was conducted using iProof HF Master Mix (Bio-Rad) according to the supplier’s recommendations and using in house gDNA, except for *Fusarium oxysporum* which was obtained through gene synthesis (GeneArt™, Regensburg, Germany), based on mitochondrial genome sequence from *F. oxysporum* strain UASWS AC1 (GenBank KR952337.1). The PCR products were purified using QIAquick PCR purification kit (Qiagen, Venlo, The Netherlands) and eluted in 30 µL of RNAse-free water.

#### 4.2.2. In Vitro Transcription

The PCR products were transcribed for 6 h at 37 °C using MegaScript kit (Ambion, Kaufungen, Germany), digested for 5 min at 37 °C with TURBO DNAse (Ambion) and purified using MegaClear kit (Ambion). The purified transcription products were precipitated for 16 to 18 h at −20 °C in 100% EtOH, 10% (*v*/*v*) 3.0 M sodium acetate (pH 5.3), and resuspended in RNAse-free water [20]. RNA concentration and purity were assessed using NanoDrop OneC and the profiles were observed after denaturing gel electrophoresis using a 10% Mini-PROTEAN^®^ TBE-Urea Precast gel (Bio-Rad, Hercules, CA, USA) or using the Experion automated electrophoresis system with the RNA StdSens analysis kit (Bio-Rad) after a denaturation at 90 °C for 2 min followed by 5 min in ice.

### 4.3. Splicing Assays and Splicing Products Quantification

#### 4.3.1. Splicing Assays

Self-splicing reactions proceeded in two steps. First, produced RNA were incubated in a buffer containing 25 mM NaCl, 15 mM MgCl_2_, 25 mM HEPES (pH 7.5) [19] at 60 °C for 3 min, followed by a slow temperature decrease to 37 °C afterwards. Then, self-splicing reactions were run in 10 µL mixes containing 100 nM RNA and 0.6 mM GTP and incubated at 37 °C for 1 h. Buffer and salts were then removed using RNA Clean & Concentrator kit (Zymo Research, Irvine, CA, USA) before splicing investigations.

#### 4.3.2. Primers Used for RT-PCR and RT-qPCR Analysis

F1 and R1 primers corresponded to the 5′ splice site sequence and amplified only pre-RNA cDNA (Figure 1; Table S3). F3 and R3 corresponded to the 3′ splice site sequence and amplified pre-RNA and G-intron-5′ exon cDNA. F2 and R2 corresponded to the group I intron and were used to assess nucleic acid degradation when compared to F1/R1 and F3/R3. F1 and R3 corresponded to the ligation of the 5′ exon-3′ exon and amplified only this cDNA splicing product.

#### 4.3.3. Self-Splicing Direct Visualization and Relative Quantification

All splicing reactions were visualized using the Experion automated electrophoresis system with the RNA StdSens analysis kit (Bio-Rad). Reverse transcriptions (RT) for PCR and qPCR were performed with 500 ng of RNA and 2 pmole of R3 specific primer described earlier, using SuperScript™ II RT (Invitrogen, Karlsruhe, Germany) and run as described by the manufacturer. Quantitative RT-PCR (RT-qPCR) were conducted with the LightCycler^®^ 480 Instrument II (Roche, Basel, Switzerland) and the SsoAdvanced™ Universal SYBR^®^ Green Supermix (Bio-Rad) following the manufacturer’s recommendations. The RT-qPCR was followed by a melting curve analysis and the size of the product was confirmed by electrophoresis on agarose gel. The relative amount of splicing products (Pre-RNA, G-intron-3′ exon and 5′ exon-3′ exon) was determined using an adaptation of the 2^−ΔΔCt^ method [68]. Briefly, an arbitrary value of 1 was given to the pre-RNA amount. The pre-RNA and G-intron-5′ exon amounts were calculated with 2^(F1/R1–F3/R3)^, so G-intron-5′ exon corresponded to 1–2^(F1/R1–F3/R3)^. Finally, 5′ exon-3′ exon was estimated with 2^(F1/R1–F1/R3)^ and all splicing products were represented as a percentage of the quantified RNA population.

RT-PCR were conducted using RT-qPCR primers and the PCR method described above with 1 ng of cDNA as template. RT-PCR products were checked on agarose gels and by Sanger sequencing (Eurofins Genomics, Ebersberg, Germany).

### 4.4. Secondary Structure Predictions and Ribozyme Construction

Secondary structure diagrams were adapted from a similar intron in *Podospora anserina* (Figure S4), described on the Gutell Lab’s Comparative RNA Website [69]. Based on the secondary structure prediction, the ribozyme was constructed as followed: a 5′ Duplex domain was added in 5′ (5′-TCATCCTTACATATTCAACACATAACCAAT) followed by the group I intron (starting at the second half of the P1 domain, underlined) and 80 bp of the 3′ exon [12,21]. The PCR product was then cloned into a pCR4-Blunt-TOPO plasmid using the Zero Blunt PCR Cloning kit (Invitrogen) and a PCR-mediated plasmid DNA deletion was performed to obtain the 4-bp deletion in the P7 domain for the deleted ribozyme. The deleted bases (5′-CAGA-3′) are written in bold red (Figure S9).

The substrate is composed of the first half of the P1 domain (underlined), pursued by the reverse-complement of the 5′ Duplex (5′-TGGGTTACCATTGTTGAATATGTAAGGATGA-3′). RNA binding free energies were computed with IntaRNA, from Vienna RNA package [70]. The ribozyme was produced from a PCR template as described earlier for the native intron and the unlabeled substrate was ordered as a single strand RNA (ssRNA) from Eurofins Genomics.

### 4.5. Trans-Splicing Validation

#### 4.5.1. Trans-Splicing Fluorescent Assay

RNA substrate carries a 5′ IowaBlack^®^ Quencher (in bold) and Internal 6-FAM Fluorophore within the P1 loop (underlined): 5′-**U**GGGUUACCAUUGUUGAAUAUGUAAGGAUGA-3′ and were ordered from IDT (Integrated DNA Technologies, Coralville, IA, USA). RNA substrate was dissolved in 5 mM HEPES, 20 mM KCl and 0.2 mM MgCl_2_ (pH 7.4) at 100 µM (stock solution) and aliquoted at 1 µM (working solution) in nuclease-free water. Each *trans*-splicing reaction was composed of 50 nM substrate, 100 nM ribozyme, 0.6 mM GTP if added and a buffer containing 25 mM NaCl, 15 mM MgCl_2_, 25 mM HEPES (pH 7.5) [19]. 14-nt (5′-AUGGUAACCCAGGA-3′) and 35-nt (5′-UCAUCCUUACAUAUUCAACAAUGGUAACCCAGGAA-3′) antisense RNAs were transcribed and purified as described earlier using oligo duplexes from Eurofins Genomics as DNA templates. Mitoxantrone dihydrochloride (Merck, Darmstadt, Germany) was resuspended in DMSO and 0.3 µL of either DMSO or mitoxantrone (for a final concentration of 10 µM) were added in a 10 µL splicing reaction and incubated at 37 °C. FAM fluorescence was measured using QuantStudio™ 6 Flex (Applied Biosystems, Darmstadt, Germany).

#### 4.5.2. Beacon Assay

For the beacon assay, the 5′ exon-3′ exon DNA beacon probe carries a 5′ FAM fluorophore and a 3′ IowaBlack^®^ Quencher and was ordered from IDT (Integrated DNA Technologies). The beacon is composed of a stem (underlined) and a loop complementary to the target of interest (5′-CCAGGTAAAACATAACCCAGGAACCTGG-3′). The DNA beacon was dissolved in 5 mM HEPES, 20 mM KCl and 0.2 mM MgCl_2_ (pH 7.4) at 100 µM (stock solution) and aliquoted at 1 µM (working solution) in nuclease-free water. Briefly, the splicing reactions were conducted for 1 h at 37 °C as previously described but with a non-fluorescent substrate from Eurofins Genomics (5′-UGGGUUACCAUUGUUGAAUAUGUAAGGAUGA-3′). Then, 1 µL of the 5′ exon-3′ exon DNA beacon was added to 9 µL of the splicing reaction for a final concentration of 50 nM. The reaction was then incubated at 70 °C for 5 min and at 37 °C for 20 min prior reading. FAM fluorescence was measured using QuantStudio™ 6 Flex (Applied Biosystems).

#### 4.5.3. Statistical Analyses

Statistical analyses were conducted on R (v. 4.0.1) using a multi-factor ANOVA followed by a Bonferroni correction of the *p*-values to control the family-wise error rate. For the beacon assay (Figure 3B), the mean is defined as a GTP, ribozyme, substrate, and batch effects. The experiment is composed of three biological replicates, with three technical replicates per condition. If the adjusted *p*-value was lower than 0.01, the difference was considered significant.

## Data Availability

The data presented in this study are available on request from the corresponding author.

## Supplementary Materials

The following supporting information can be downloaded at https://www.mdpi.com/article/10.3390/molecules28114460/s1: Figure S1: In vitro self-splicing activity of 10 fungal group I introns, assessed by automated electrophoresis (Experion); Figure S2: Quantitative Reverse Transcription PCR (qRT-PCR) of reaction products before and after splicing for *Botrytis cinerea* cob (4) and *Phakopsora pachyrhizi* cob (1); Figure S3: Sanger sequencing result of *Fusarium cob* 5′Exon-3′Exon after in vitro cis-splicing and RT-PCR aligned with expected sequence; Figure S4: *Podospora anserina* cob secondary structure prediction from the Gutell Lab’s Comparative RNA Website; Figure S5: Putative splicing mechanism of *Fusarium* cob (1) intron; Figure S6: Sanger sequencing result of *Fusarium cob* 5′Exon-3′Exon after in vitro *trans*-splicing and RT-PCR aligned with expected sequence; Figure S7: Comparison between fluorescent substrates used in this work; Figure S8: Fluorescence signal before and after 1 h at 37 °C; Figure S9: Secondary structure prediction of *Fusarium oxysporum* cob [i1]; Table S1: List of fungal Group I introns tested in this work; Table S2: List of primers used for T7 PCR amplification; Table S3: List of primers used for qRT-PCR.

## Sample Availability

DNA templates for in vitro transcription are available from the authors.
