# Supplementary material for: Group I Intron as a Potential Target for Antifungal Compounds: Development of a Trans-Splicing High-Throughput Screening Strategy
Source: Molecules. 2023 May 31;28(11):4460. doi: 10.3390/molecules28114460 (PMC10254303; doi:10.3390/molecules28114460)
Supplement: Supplementary file 1 [file molecules-28-04460-s001.zip › molecules-2404624-supplementary.pdf]

Botrytis cinerea<br>cob (2)<br>ID<br>Not detected	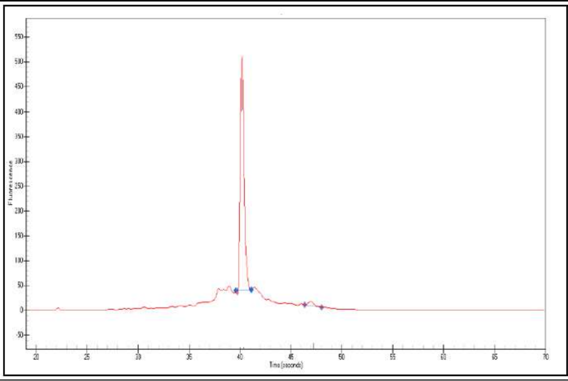	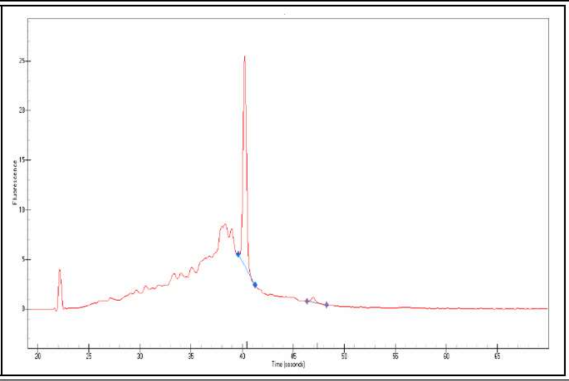
Botrytis cinerea<br>cob (4)<br>IA<br>Partial	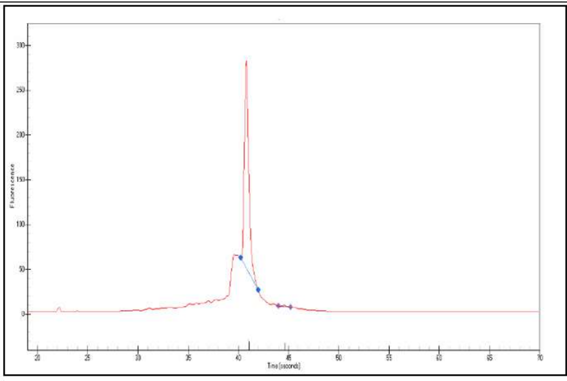	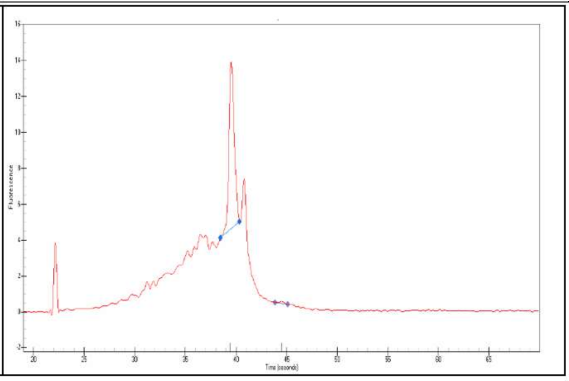
Fusarium oxysporum<br>cob (1)<br>ID<br>Detected	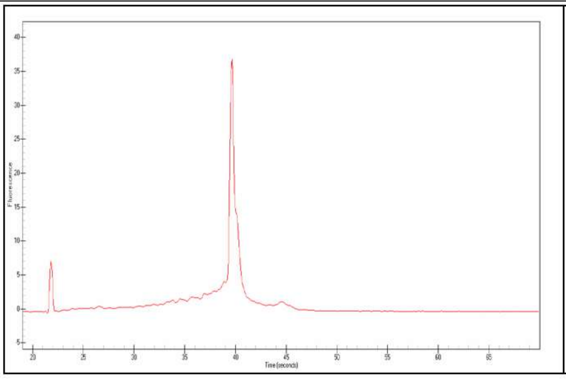	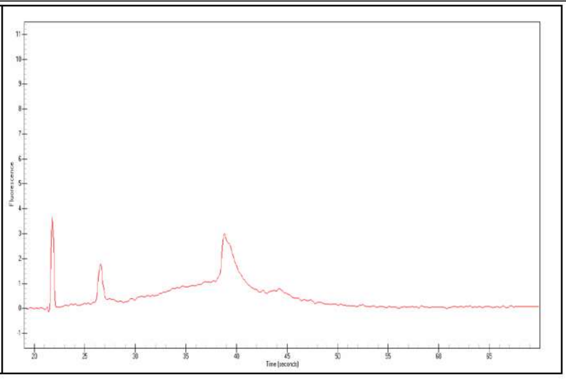
Phakopsora pachyrhizi<br>cob (1)<br>ID<br>Partial	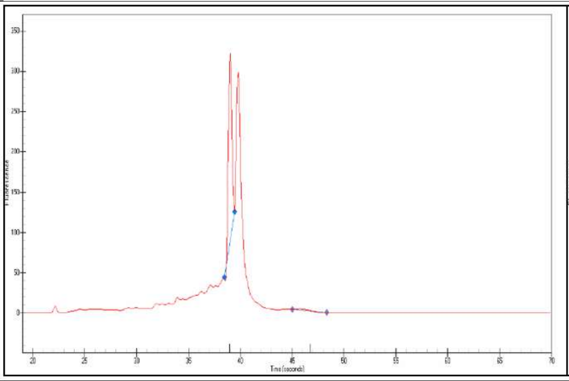	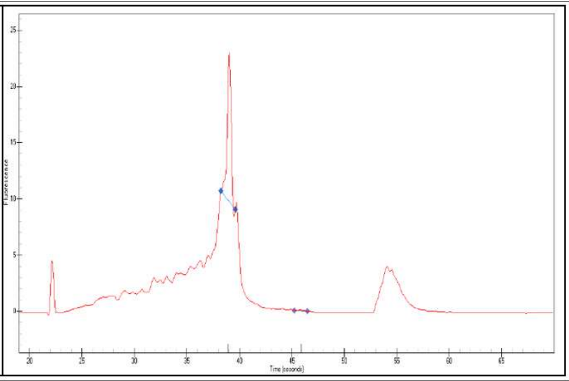
Phakopsora pachyrhizi<br>cox1 (1)<br>IB<br>Not detected	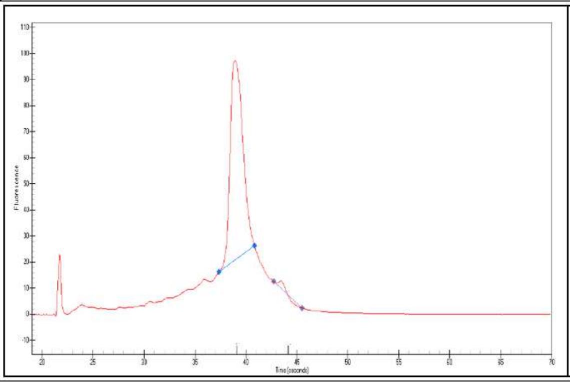	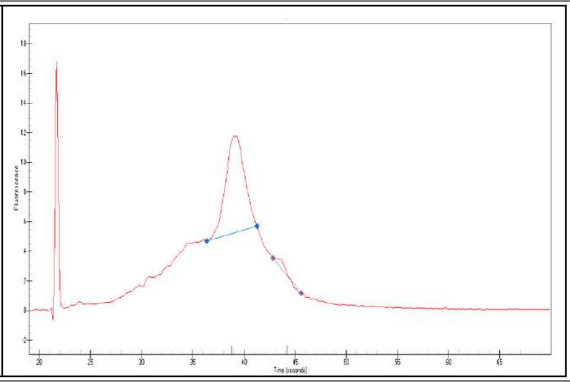

Phakopsora pachyrhizi<br>cox1 (2)<br>ID<br>Not detected	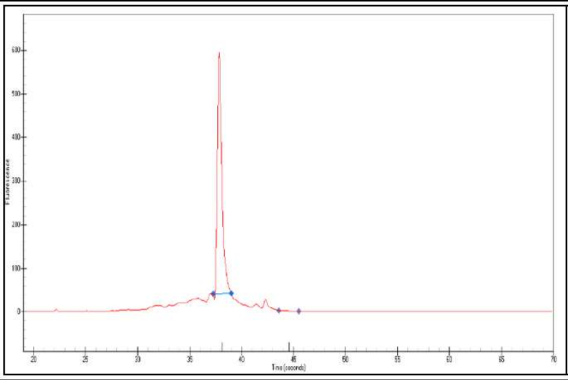	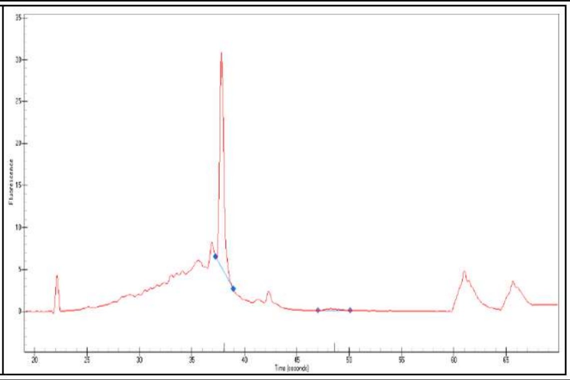
Phakopsora pachyrhizi<br>rnl (1)<br>IB<br>Not detected	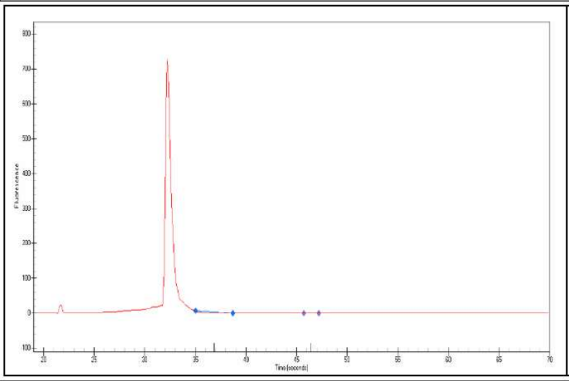	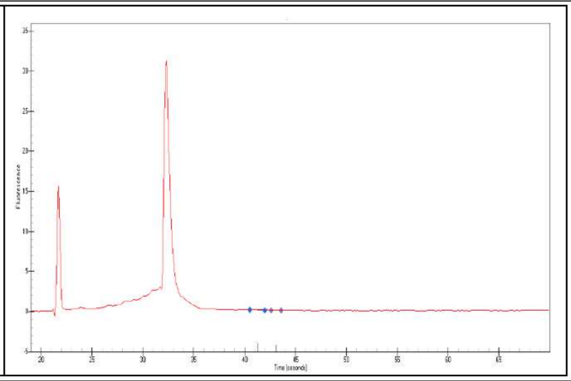
Ustilago maydis<br>cox1 (4)<br>ID<br>Not detected	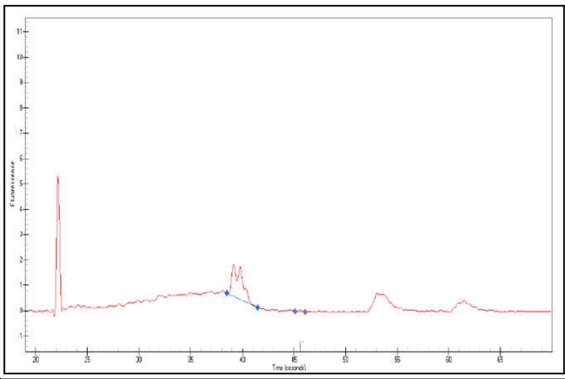	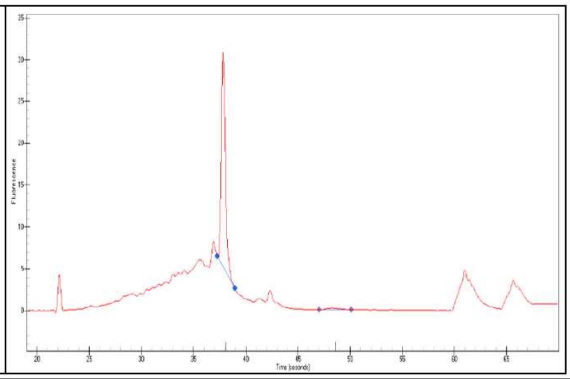
Ustilago maydis<br>cox1 (6)<br>IB<br>Not detected	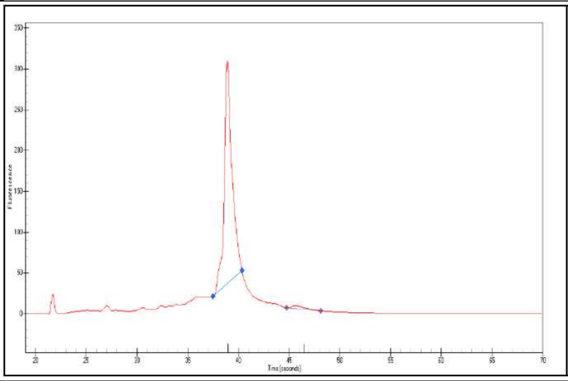	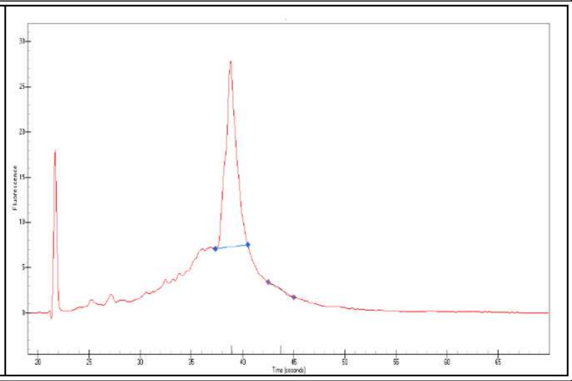
Ustilago maydis<br>cox1 (8)<br>IA<br>Not detected	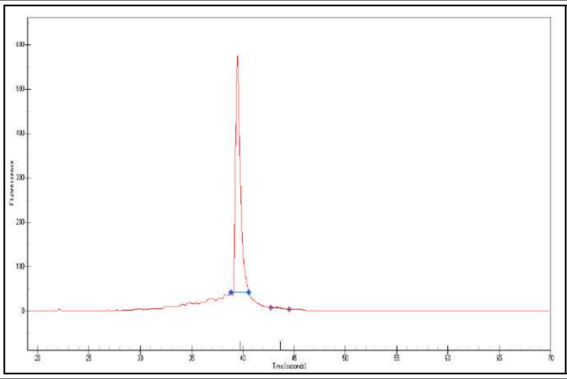	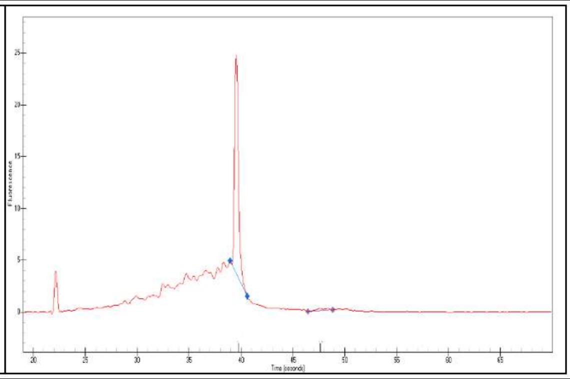

Figure S1: *In vitro* self-splicing activity of 10 fungal group I introns, assessed by automated electrophoresis (Experion). From left to right, before and after self-splicing reactions.

A

B

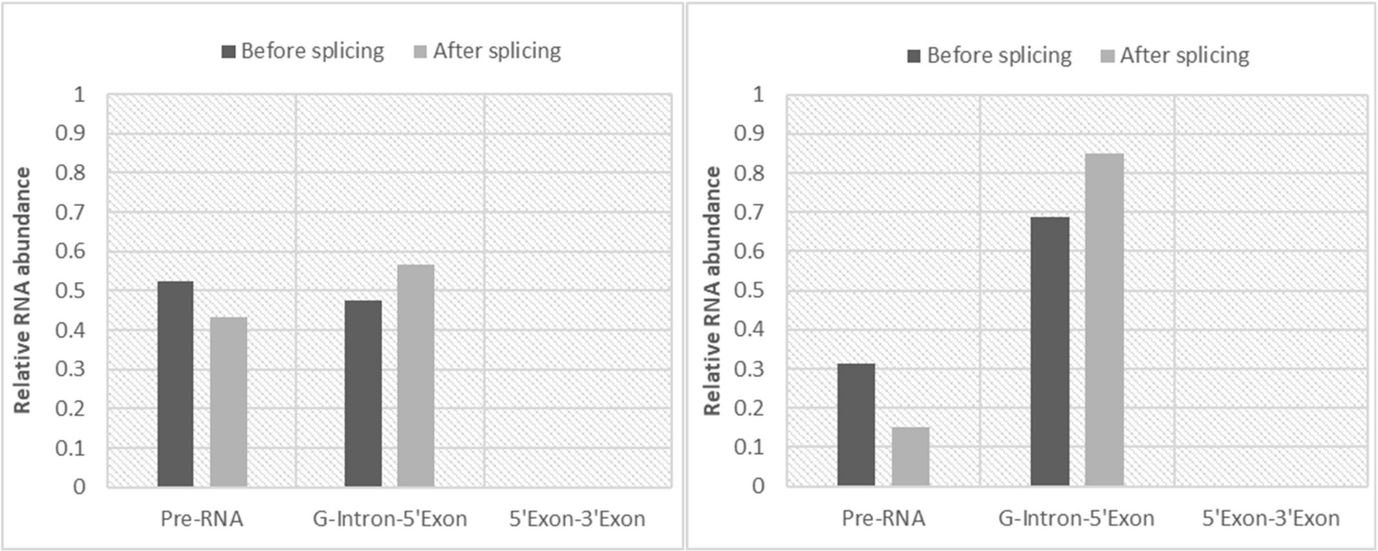

Figure S2: Quantitative Reverse Transcription PCR (qRT-PCR) of reaction products before and after splicing for (A) *Botrytis cinerea* cob (4) and (B) *Phakopsora pachyrhizi* cob (1). Representative result of three independent experiments, based on three technical replicates.

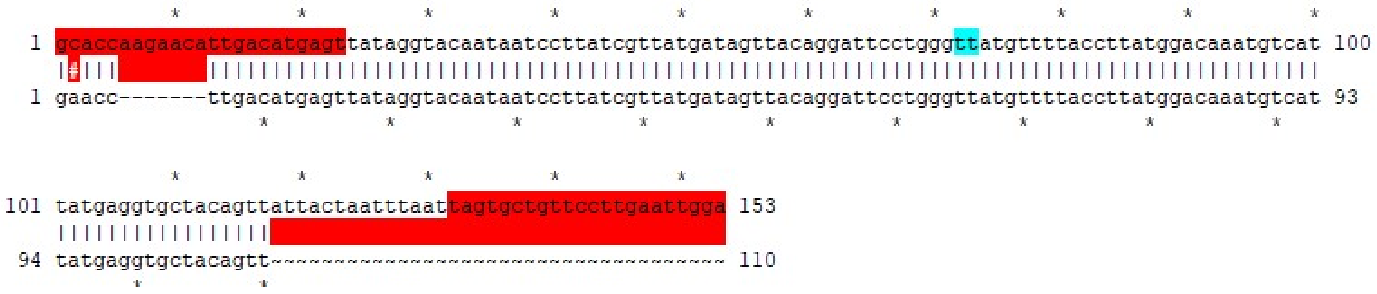

Figure S3: Sanger sequencing result of *Fusarium* cob 5’Exon-3’Exon after *in vitro* cis-splicing and RT-PCR aligned with expected sequence.

On top, the expected 5’Exon-3’Exon sequence with forward and reverse primers used for RT-PCR in red and splice site in blue. Below, Sanger sequencing result using the reverse primer for amplification.

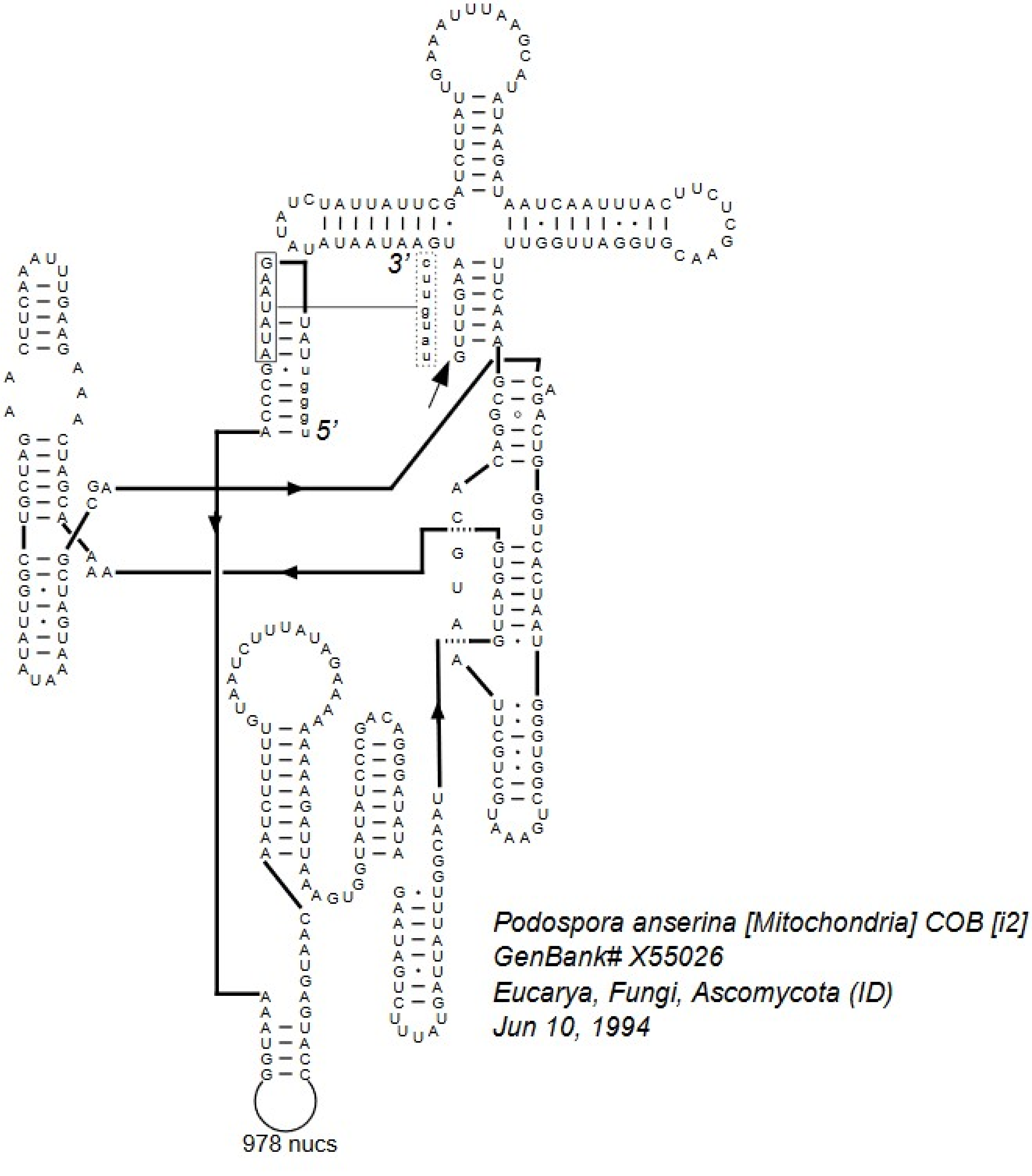

Figure S4: *Podospora anserina* cob secondary structure prediction from the Gutell Lab’s Comparative RNA Website (https://crw-site.chemistry.gatech.edu/).

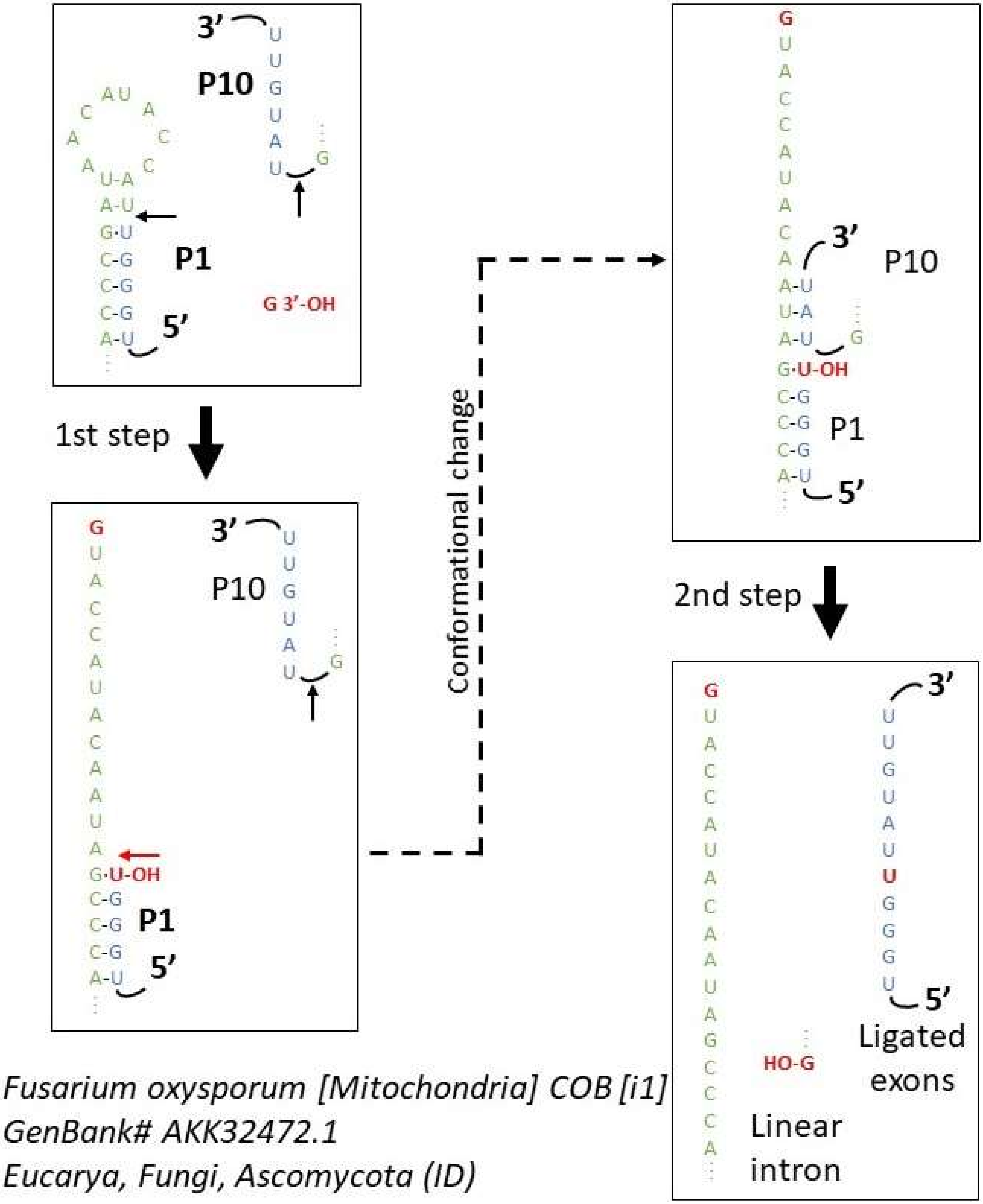

Figure S5: Putative splicing mechanism of *Fusarium* cob (1) intron (inspired from Vicens & Cech, 2006). The green sequence corresponds to the intron, the blue sequence to the exons, the red sequence to the guanosine co-factor and the black arrow to the splice sites.

```
        *           *           *           *           *           *           *           *           *
 1 gtgcatgcgttcctgggTTatgttttaccttatggacaaatgtcattatgaggtgctacagttattactaatttaattagtgctgttccttgaattgga 99
   ||||||||||||||||||||||||||||||||||||||||||||||||||||                                           |
53 gtgcatgcgttcctgggttatgttttaccttatggacaaatgtcattatgag-------------------------------------------t~~~ 1
     *           *           *           *           *
```

Figure S6: Sanger sequencing result of *Fusarium* cob 5’Exon-3’Exon after *in vitro trans*-splicing and RT-PCR aligned with expected sequence.

On top, the expected 5’Exon-3’Exon sequence with forward and reverse primers used for RT-PCR in red and splice site in blue. An extra tail was added in 5’ of the forward primer (5’-GTGCATGCG…) completed with a specific sequence stopping before the splice site to ensure specificity (…TTCCTGGGT-3’). Below, Sanger sequencing result using the reverse primer for amplification.

A

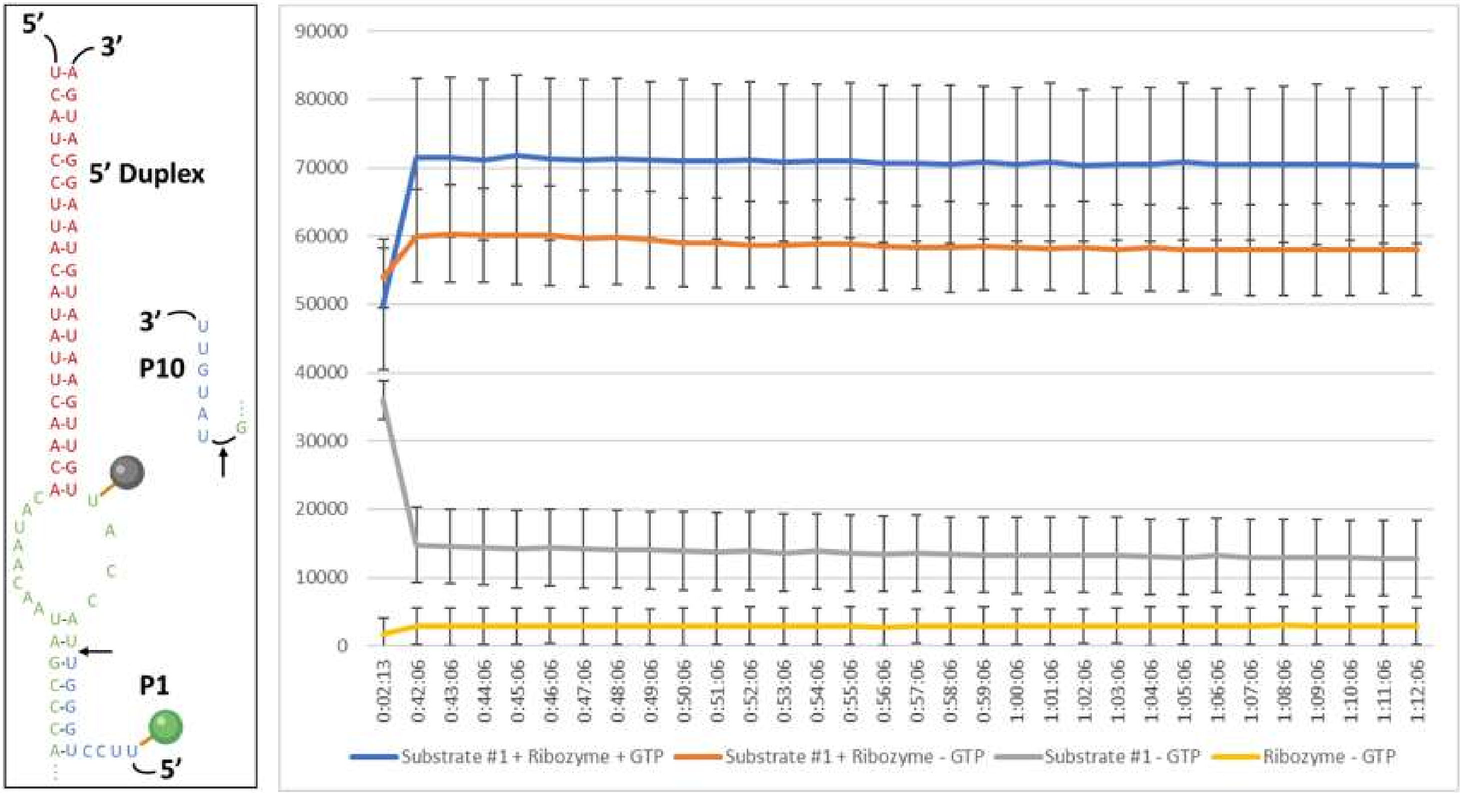

B

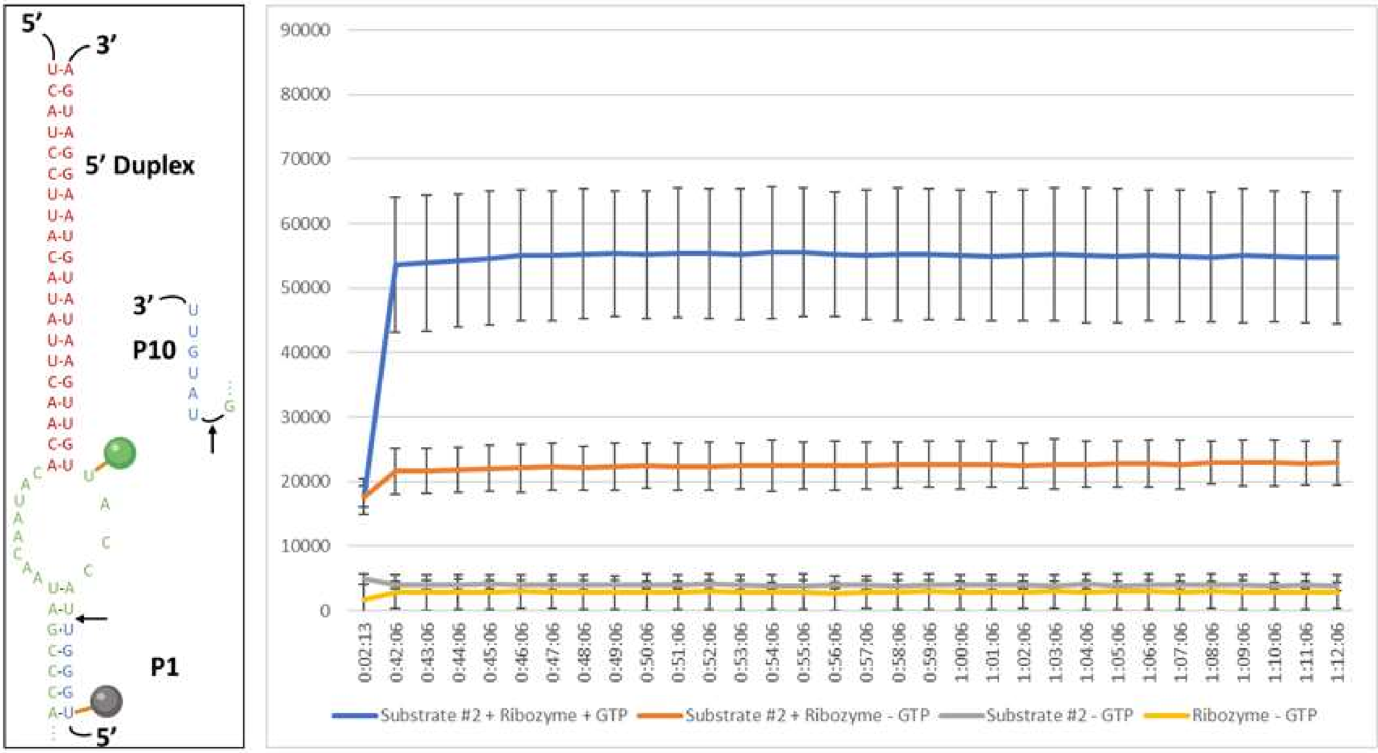
5′
3′
5′ Duplex
3′
P10
P1
5′
90000
80000
70000
60000
50000
40000
30000
20000
10000
0
Substrate #2 + Ribozyme + GTP
Substrate #2 + Ribozyme - GTP
Substrate #2 - GTP
Ribozyme - GTP

C

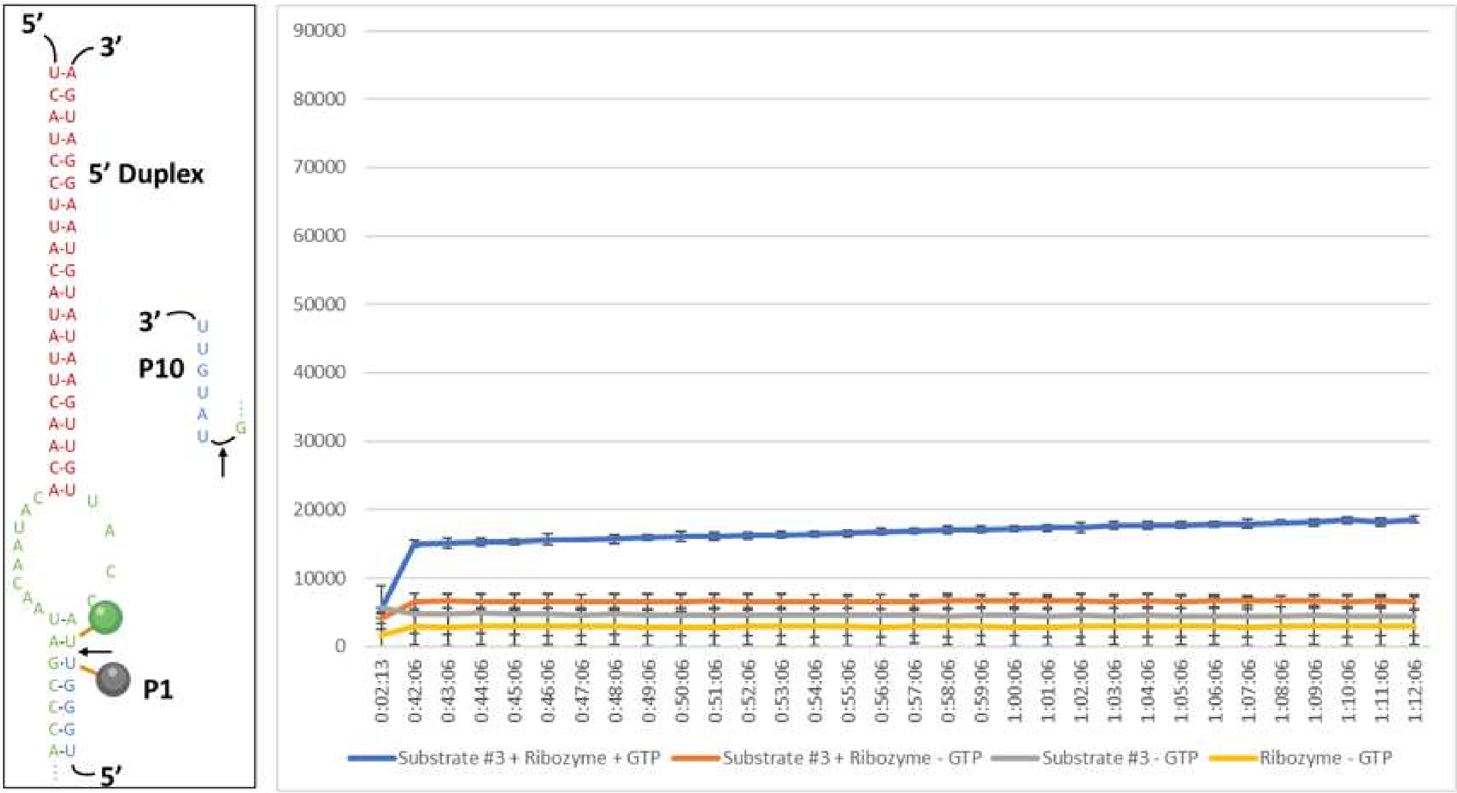
5′
3′
5′ Duplex
3′
P10
P1
5′
90000
80000
70000
60000
50000
40000
30000
20000
10000
0
Substrate #3 + Ribozyme + GTP
Substrate #3 + Ribozyme - GTP
Substrate #3 - GTP
Ribozyme - GTP

Figure S7: Comparison between fluorescent substrates used in this work. Blue line corresponds to a classical self-splicing reaction, composed of ribozyme, substrate, salts and GTP; orange line corresponds to the same reaction without GTP; grey line corresponds to substrate and salts and yellow line ribozyme and salts. (A) Substrate #1 is composed of a FAM fluorophore in green (maximum excitation: 495nm ; maximum emission: 515nm) and a DABdT quencher in grey (maximum absorbance: 475nm); (B) Substrate #2 and (C) #3 are composed of a 5' Iowa Black® Fluorescence Quencher in grey (maximum absorbance: 531nm) and an Internal-6-FAM fluorophore in green (maximum excitation: 495nm ; maximum emission: 515nm). All reactions were performed and monitored as described in "Materials and Methods". The black arrow corresponds to the splice site. Error bars correspond to standard deviation.

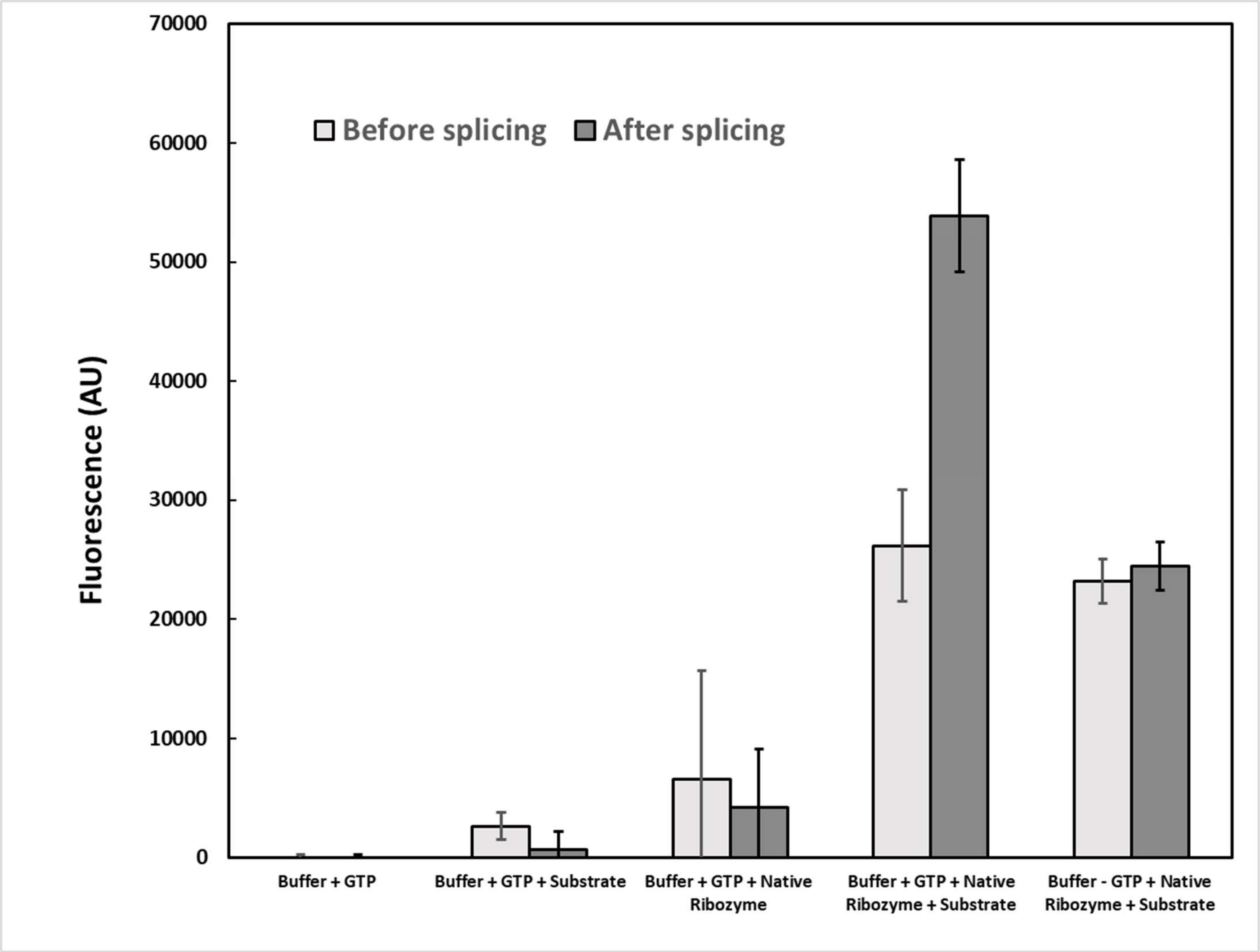

Figure S8: Fluorescence signal before (light grey) and after 1h at 37°C (dark grey). Fluorescence is only observed when the ribozyme and the substrate are co-incubated, with a twofold increase in signal under proper reaction conditions. Error bars correspond to standard deviation.

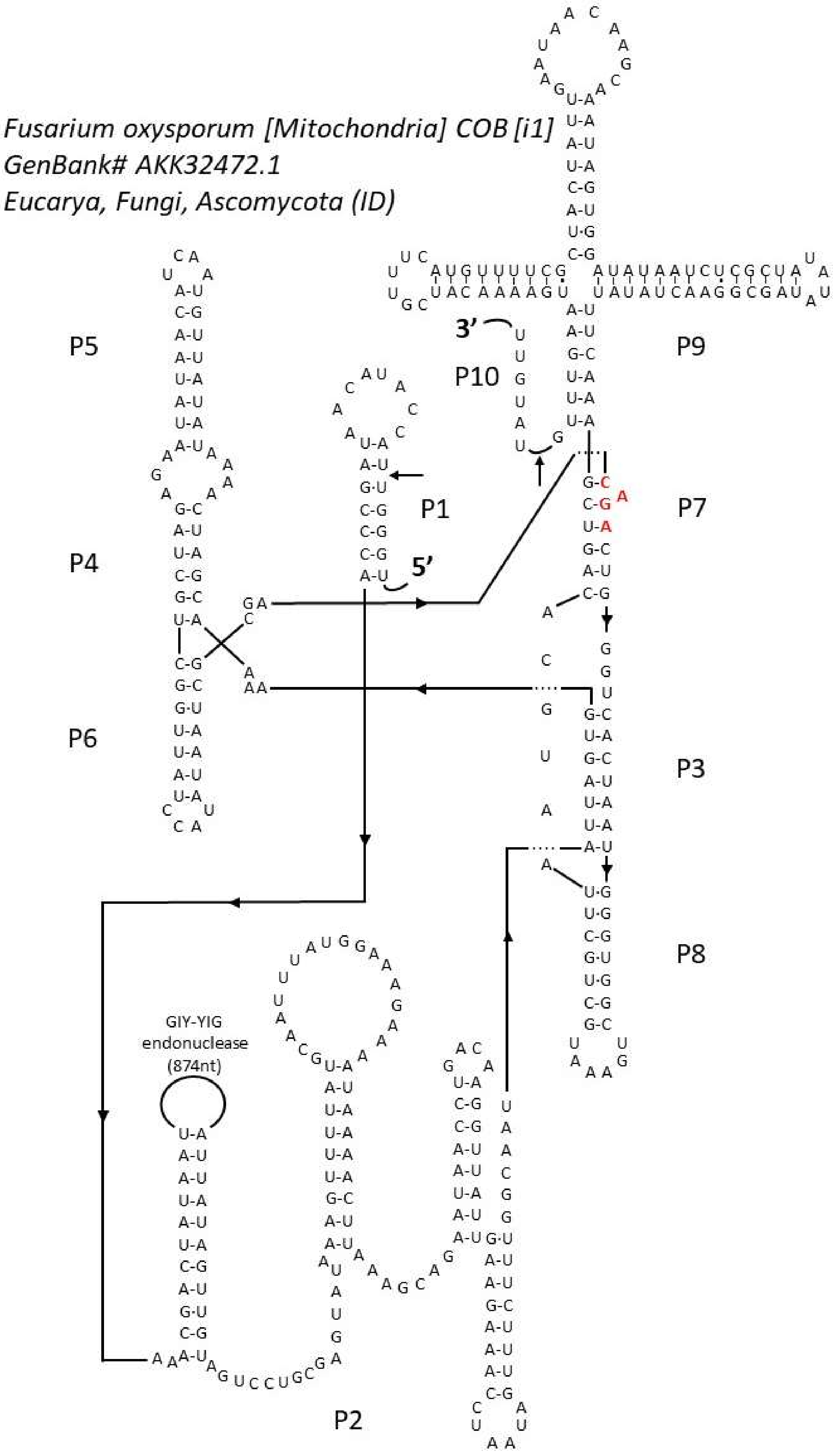

Figure S9: Secondary structure prediction of *Fusarium oxysporum* cob [i1]. The bases in bold red are deleted in the 4-nt deletion mutant used in Fig. 5.

Table S1: List of fungal Group I introns tested in this work.

Organism name	Intron location	Gene[1] (position)	Subgroup[2]	Self-splicing activity[3]	Intron length (nt)	GC content (%)	Genome Genbank
Botrytis cinerea	Mitochondrion	cob (2)	ID	Not detected	1358	28%	KC832409.1
Botrytis cinerea	Mitochondrion	cob (4)	IA	Partial[4]	1245	28%	KC832409.1
Fusarium oxysporum	Mitochondrion	cob (1)	ID	Detected	1238	27%	KR952337.1
Phakopsora pachyrhizi	Mitochondrion	cob (1)	ID	Partial[4]	1337	37%	GQ332420.1
Phakopsora pachyrhizi	Mitochondrion	cox1 (1)	IB	Not detected	1120	34%	GQ332420.1
Phakopsora pachyrhizi	Mitochondrion	cox1 (2)	ID	Not detected	935	36%	GQ332420.1
Phakopsora pachyrhizi	Nuclear	rnl (1)	IB	Not detected	366	38%	GQ332420.1
Ustilago maydis	Mitochondrion	cox1 (4)	ID	Not detected	1397	33%	DQ157700.1
Ustilago maydis	Mitochondrion	cox1 (6)	IB	Not detected	1349	31%	DQ157700.1
Ustilago maydis	Mitochondrion	cox1 (8)	IA	Not detected	1208	39%	DQ157700.1

[1]rnl: large subunit rRNA; cob: cytochrome b; cox1: cytochrome oxidase subunit 1

[2]Based on RNAWeasel predictions (Lang et al., 2007).

[3]Assessed with qRT-PCR and/or automated electrophoresis

[4]Correspond to the first step of the self-splicing reaction

Table S2: List of primers used for T7 PCR amplification.

Underlined sequences correspond to T7 promoter.

Organism name	Gene type (position)	Forward primer (5' -> 3')	Reverse primer (5' -> 3')
Botrytis cinerea	cob (2)	TAATACGACTCACTATAGGGCAGTAACACAGCTTCAGC	CTCACAGTGACATTTGCCC
Botrytis cinerea	cob (4)	TAATACGACTCACTATAGGGTTATTACAAATCTTATGAGTGCTG	GGTTACCAGAACCTGCACTATC
Fusarium oxysporum	cob (1)	TAATACGACTCACTATAGTCTTAATAGAGCACCAAGAAC	TCCAATTCAAGGAACAGCACTA
Phakopsora pachyrhizi	cob (1)	TAATACGACTCACTATAGGGCGATTGGAGTAATTATTC	TCTATTCAGAGTCGCATTACTTACACTGAAACCC
Phakopsora pachyrhizi	cox1 (1)	TAATACGACTCACTATAGGGTTTGACTGCTATGCCCG	ATCCCCGCTAGATGTAGAGAAAAAATTACACAATC
Phakopsora pachyrhizi	cox1 (2)	TAATACGACTCACTATAGGGTAACAGACAGGAATTTCAAC	ACTGCCTTACCACTAAACTG
Phakopsora pachyrhizi	rnl (1)	TAATACGACTCACTATAGGGTATAAAGTGTTTTAGG	ACTAGCATACATGTTAAGACAGCTAGAGCATC
Ustilago maydis	cox1 (4)	TAATACGACTCACTATAGGGACTCCTTAGAGCTTTTGTTAC	TGTTTTTGAGAATAGGTGTTGATAAAG
Ustilago maydis	cox1 (6)	TAATACGACTCACTATAGGGCAGGTAAACCAGTATTTG	GTAGGAACAGCAATAATCATTGTAGCAGC
Ustilago maydis	cox1 (8)	TAATACGACTCACTATAGGGAAACCATCTACCCTATCTCG	TGCAGTAGCAACTACAGATACTAG

Table S3: List of primers used for qRT-PCR.

Organism name	Gene type (position)	Name	Sequence (5' -> 3')
Fusarium oxysporum	cob (1)	F1	GCACCAAGAACATTGACATGAGT
Fusarium oxysporum	cob (1)	R1	ACGTGAGAACGAAGTCTCCT
Fusarium oxysporum	cob (1)	F2	AGTTGTAGTCCTGCGAGTATAAAG
Fusarium oxysporum	cob (1)	R2	GACCCAGTCTGTCGCGATTA
Fusarium oxysporum	cob (1)	F3	ACTAATGGGTGGCTGAAATGC
Fusarium oxysporum	cob (1)	R3	TCCAATTCAAGGAACAGCACTA
Botrytis cinerea	cob (4)	F1	TGAGTGCTGTACCATGAATTGGA
Botrytis cinerea	cob (4)	R1	ACCTATTAGAGGTAGCATCGTTAAA
Botrytis cinerea	cob (4)	F2	GAGTGGGGTTCACAATGGCT
Botrytis cinerea	cob (4)	R2	ACTCTTTGTACGTCGGCCTT
Botrytis cinerea	cob (4)	F3	CTCGACACGAATGCCCTACA
Botrytis cinerea	cob (4)	R3	AAGCCGCTAAAACGAAAGGT
Phakopsora pachyrhizi	cob (1)	F1	AGCGACAGCGTTTATAGGTT
Phakopsora pachyrhizi	cob (1)	R1	ACTGCATGTGAGGCGGTC
Phakopsora pachyrhizi	cob (1)	F2	TTTGCTTTTCTCAAGCCGGG
Phakopsora pachyrhizi	cob (1)	R2	TAGCGATCACTCACCCCTCT
Phakopsora pachyrhizi	cob (1)	F3	CAGGGTCATGTATTGGGGGC
Phakopsora pachyrhizi	cob (1)	R3	TCCAATTCAGGGAATCGCACT
